# Nuances of reproductive decisions by women in a rural community of Lagos, Nigeria

**DOI:** 10.11604/pamj.2020.37.133.18235

**Published:** 2020-10-06

**Authors:** Kikelomo Ololade Wright, Omowunmi Bakare, Adeyinka Adeniran, Modupe Akinyinka, Yetunde Kuyinu, Olayinka Goodman

**Affiliations:** 1 Department of Community Health and Primary Health Care, Lagos State University College of Medicine, Ikeja, Lagos, Nigeria

**Keywords:** Reproductive health, decision-making, maternal health, spouse

## Abstract

**Introduction:**

inadequate utilization of maternal health services due to limited reproductive decision-making capacity could be contributory to high maternal mortality in developing countries. This study sought to assess nuances of reproductive decisions by women in a rural community of Lagos, Nigeria.

**Methods:**

this descriptive, cross-sectional house to house survey was part of a study conducted in April 2015 on females selected from 298 households chosen based on geographical clusters by simple random sampling. The study instrument was adapted from a USAID-funded project and was interviewer-administered. Data entry and analysis were performed with the aid of Epi-info™ 7.0.8.3 statistical software and ethical approval was obtained for the study.

**Results:**

spousal age difference was less than 10 years for about half (51.3%) of the respondents. The majority (91.6%) of the respondents had received antenatal care during pregnancy and jointly decided with their spouses on place of care. The most commonly used contraceptives were the pills (23.5%), injectables (16.8%) and condoms (13.8%). Spousal disapproval regarding the use of family planning was almost nil at 1%. Employment status as a socio-economic factor did not significantly affect respondents´ involvement in decision-making. However, there were statistically significant associations between spousal age differences and some indicators of autonomy such as respondents´ involvement in health care decisions and the determinant on choice of antenatal care provider.

**Conclusion:**

women´s reproductive independence and involvement in health decisions could result in reduction of maternal ill-health and mortality whilst promoting higher male involvement and better maternal health.

## Introduction

Maternal and child mortality have remained as major health problems in developing countries. According to the World Health Organization (WHO), an estimated 358,000 maternal deaths occur globally on an annual basis and about 99% of these cases are seen in developing countries, with sub-Saharan Africa accounting for 57% of these deaths [1]. An assessment of the Millennium Development Goals (MDGs) by the United Nations revealed an estimated 45% reduction in global maternal mortality ratio (MMR) between 1990 and 2015 [2]. Even though projections from the report imply that the goal of a 75 percent reduction by 2015 may not have been attainable, the improvement was notable. This goal is intensified by the Sustainable Development Goals (SDGs) which seek to build on previous initiatives aimed at reducing MMR from preventable causes to less than 70% by 2030 [3]. Achieving the SDGs on maternal health requires a multi-pronged approach geared towards the provision of quality care during pregnancy and delivery, essential obstetric care services and improving women´s sexual and reproductive health, enabling fulfilled sexuality with the capacity to determine the timing and frequency of reproduction [3,4].

Reproductive health promotion strategies for women range from efforts to strengthen health service delivery in terms of access to emergency obstetric care, improving coverage and quality of skilled attendance at birth, post-abortion care, revamped reproductive health services for adolescents and enhanced family planning services. Of paramount importance is the health care seeking behavior of women which is pertinent in the prevention and control of morbidity related to reproductive health [5,6]. According to the World Bank, Nigeria´s MMR of an estimated 560 deaths per 100,000 live births is one of the highest in the world. Nonetheless, an estimated 800 women die daily from complications related to childbirth [4]. It may appear that inadequate utilization of reproductive health services in Nigeria could be contributory to the high maternal mortality figures observed. The 2013 Nigerian Demographic and Health Survey (NDHS) reported that only an estimated 15% of married women were practicing family planning at the time. Even though 61% had received antenatal care for their most recent birth, skilled attendance at delivery was found in 38% of cases [7].

Research has shown that the utilization of reproductive health services can be influenced by a multiplicity of factors such as socio-demographic characteristics and women´s autonomy within the household [8]. The ability to not only recognize signs inimical to wellbeing but also actively take health care decisions, has the potential of reducing adverse reproductive outcomes amongst women. Given the gains in the reduction of inequities and provision of reproductive health services globally, women in rural low-resource settings are often disadvantaged. According to an Indian study, indicators such as women´s decision making capacity, education, employment, access to media and spousal age difference are capable of influencing women´s health-seeking behavior [9]. Other reported determinants of women´s autonomy include access to and control over resources, demonstrated participation in economic decisions, self-esteem, mobility, and freedom from domestic violence [10,11]. Factors such as attitude towards wife beating, right to refuse sex and permission to seek healthcare have been acknowledged as determinants for the utilization of reproductive health services which include family planning services and antenatal care [12].

Women´s autonomy in health care decisions is extremely important for better maternal and child outcomes, more so in rural communities, which are often characterized by sub-optimal health statistics [13]. This study is part of a quest to better understand reproductive health autonomy and nuances of reproductive decisions by women in a rural community of Lagos, Nigeria.

## Methods

Agbowa-Ikosi is a largely rural community in Lagos State, Nigeria divided into wards. The town is headed by a recognized traditional ruler alongside other chiefs and the main occupations include farming, fishing and trading in food crops such as cassava and maize. The community consists of several primary and secondary educational institutions provided by the government and private organizations. It is served by a relatively functional public transport system and a reasonable road network. Reproductive health care services are provided by primary and secondary government health institutions (a general hospital and four primary health centres), private health practitioners and traditional birth attendants (TBAs). This descriptive, cross-sectional house to house survey on nuances of reproductive decisions was conducted from 2^nd^ to 19^th^ June 2015 as part of a comprehensive study on reproductive health autonomy amongst consenting women, 18 years and above within the reproductive age group using a pre-tested interviewer administered questionnaire adapted from a USAID funded project [14,15].

The study instrument assessed nuances of respondents´ decision making and reproductive health experiences. One female per household was selected from a total of 298 households chosen within the study period based on geographical clusters by simple random sampling. Data collection was carried out by trained medical students undergoing rural posting over a three-week period in the community. Data entry and analysis were performed with the aid of Epi-info™ 7.0.8.3 statistical software. Univariate and bivariate analyses were conducted and appropriate statistical tests of significance were performed using a 95% confidence interval and level of significance set at P<0.05. Ethical approval was obtained from the health research and ethics committee of the Lagos State University Teaching Hospital whilst permission was sought from the supervising authorities and informed consent also obtained from respondents prior to administration of the questionnaires.

## Results

Most of the respondents 128 (43.0%) were within the 30 - 39 age group, married (88.6%) and mostly (72.8%) self-employed. Age difference between respondents and their spouses was less than 10 years for about half (51.3%) of the respondents. The mean age was 31.17+7.45 (Table 1). The majority (83.6%) of respondents participated in decision-making processes at the home front and about 11.4% reported lack of spousal support for respondents´ utilization of healthcare services (Table 2). The majority (91.6%) of the respondents had received antenatal care mostly (38.4%) in primary health care centers and jointly decided with their spouses on choice of care in 39.6% of cases. The first booking/antenatal care registration was predominantly (49.1%) within the 2^nd^-3^rd^ months of pregnancy. Over 50% of the respondents registered because of the perceived importance of antenatal care and physicians were the most common category of ANC provider sought (Table 3). The most popular contraceptives used were the pills (23.5%), injectables (16.8%) and condoms (13.8%). Child spacing was the most popular reason (31.5%) for usage of family planning (FP) commodities whilst spousal disapproval regarding the use of FP was almost nil at 1% (Table 4).

**Table 1:** socio-demographic characteristics of the respondents

Variables	Frequency (N=298)	Percentage (%)
**Age group (years)**		
18-29	120	40.3
30-39	128	43.0
40-49	5	16.8
**Marital status**		
Single	21	7.0
Married	264	88.6
Separated	7	2.3
Divorced	5	1.7
Widowed	1	0.3
**Educational level**		
No formal	11	3.7
Primary	39	13.1
Secondary	216	72.5
Tertiary	32	10.7
**Employment status**		
Applicant	8	2.7
Employed	44	14.8
Home maker	8	2.7
Self employed	217	72.8
Student	21	7.0
**Religion**		
Christianity	176	59.1
Islam	116	38.9
Traditional	6	2.0
**Spousal age difference (years)**		
<10	153	51.3
>10	117	39.3
Don't know	28	9.4

**Table 2:** decision-making (DM) and denial of conjugal rights

Variable	Frequency (No)	Percentage (%)
**Participation in DM**		
Yes	249	83.6
No	49	16.4
**Spousal support for utilization of healthcare**		
Yes	264	88.6
No	34	11.4

**Table 3:** respondents´ experience with antenatal care (ANC)

Variables	Frequency (No)	Percentage (%)
**Ever received ANC**		
Yes	273	91.6
No	25	8.4
**Place of 1^st^ ANC**		
Primary health centre	105	38.4
General hospital	70	25.6
TBA	71	26.1
Private	27	9.9
Total	273	100.0
**Decision maker on choice of 1^st^ ANC**		
Respondent	76	27.8
Husband	74	27.1
Joint decision	108	39.6
Mother-in-law	15	5.5
Total	273	100.0
**First booking appointment/registration**		
2-3 months	134	49.1
4-6 months	111	40.6
>7 months	19	7.0
Don't know	9	3.3
Total	273	100.0
Reasons for seeking ANC*		
For health problems	54	19.8
Ascertain pregnancy	32	11.7
ANC is crucial	159	58.2
To check fetus safety	120	44.0
Opinion of husband/family/friend	39	14.3
Professional category of ANC providers *		
Physician	135	49.5
Nurses/midwives	93	34.1
TBA	71	26.0

* Multiple responses

**Table 4:** respondents´ experience with family planning (FP)

Variable	Frequency	Percentage
Family planning methods ever used*		
Pills	70	23.5
Injectable	50	16.8
Condom	41	13.8
Prolonged breastfeeding	28	9.4
Coitus interruption	18	6.0
Safe period	17	5.7
Traditional methods	8	2.7
IUD^	3	1.0
Subdermal implants	2	0.7
Reasons for using FP*		
Break before next pregnancy/spacing	94	31.5
Completed family	48	16.1
Desire for small family size	46	15.4
Care for self	25	8.4
Family financial status	20	6.7
Spousal decision	10	3.4
Poor health	1	0.3
Reasons for non-usage of FP*		
Wish for more children	42	14.1
Lack of information on FP	20	6.7
Temporary break	7	2.3
Spousal disapproval	3	1.0
Pregnancy after delivery	1	0.3

^Intra-uterine device

Level of education was significantly associated with respondents participation in decision making at the family level (p=0.02). It was observed that participation was more with increasing educational attainment. However, a socio-economic factor such as employment status did not significantly affect respondents´ involvement in decision making (P=0.08; Table 5). There were statistically significant associations between spousal age differences and some indicators of autonomy such as respondents´ involvement in family decisions and the determining authority on choice of antenatal care provider (P<0.05). Respondents with spousal age difference of less than 10 years were more involved in family decisions. Also, participants with spousal age difference of less than 10 years had more influence regarding the decision on choice of antenatal care providers. However, there was no statistically significant association between spousal age difference and respondents´ right to refuse conjugal intercourse for any reason (Table 6).

**Table 5:** socio-economic characteristics and participation in decision making

Decision making participant				
	Yes	No	Statistic	p-value
Variable	No. (%)	No. (%)	X^2^	
**Education**				
No formal	9 (81.8)	2 (18.2)	10.3	0.02
Primary	28 (71.8)	11 (28.2)		
Secondary	180 (83.3%)	36 (16.7)		
Tertiary	32 (100.0)	0 (0.0)		
**Employment status**				
Applicant	5 (62.5%)	3 (37.5%)	8.21	0.08
Employed	36 (81.8)	8 (18.1)		
Home-maker	7 (87.5)	1 (12.5)		
Self-employed	187 (86.2)	30 (13.8)		
Student	14 (66.7)	7 (33.3)		

**Table 6:** relationship between spousal age gap and some autonomy indicators

Variable	<10 years	>10 years	Don’t know	X
	No (%)	No (%)	No (%)	P-value
**Involved in decision making**				
Yes	132 (53.0)	100 (40.2)	17 (6.8)	11.8
No	21 (42.9)	17 (34.7)	11 (22.4)	0.003
**Decision make: choice of ANC**				
Self	51 (67.1)	24 (31.6)	1 (1.3)	
Husband and self (joint)	49 (45.4)	52 (48.1)	7 (6.5)	58.6
Husband	36 (48.7)	31 (41.9)	7 (9.4)	0.000
Mother/mother-in-law	8 (53.3)	6 (40.0)	1 (6.7)	
Others	9 (36.0)	4 (16.0)	12 (48.0)	
**Right to refuse conjugal sex**				
Yes	67 (49.6)	59 (43.7)	9 (6.7)	1.73
No	86 (57.0)	58 (38.4)	7 (4.6)	0.42

## Discussion

Women´s participation in household health care decisions is cardinal for better maternal and child health outcomes while also serving as an indicator of women empowerment. Over 80% of the participants claimed involvement in decision-making processes regarding their reproductive health. The majority of the respondents were young and within the age bracket of 18 - 39 years comparing favourably to a similar study on role of gender empowerment on reproductive health outcomes conducted in six urban Nigerian cities where more than half of the respondents were less than 35 years [16]. The high proportion of respondents with secondary or high school education and above in this study may account for the active role of participants in negotiating decisions with their spouses regarding reproductive health matters such as choice of care. This pattern varies from a demographic Nepali study on women´s autonomy where rural women had less influence on decision-making processes and outcomes such as owning health care, major household purchases and visits to family or relatives [17].

The proportion of respondents (83.6%) who reported involvement in household decision may explain the high uptake of reproductive services such as antenatal care (91.6%) and contraceptives as measures of autonomy and independence. The advantage of joint reproductive decision-making is often hinged on communication and negotiation between couples [18]. Other dimensions of women autonomy include self-determination of fertility, movement or permission to go out, domestic freedom, financial or economic self-sufficiency and choices about sexual decisions [19-21]. About half of the respondents in the other related study had opined that their spouses should not be denied conjugal sex [14]. This pattern of limited choices in sexual decisions appears commoner in developing countries on the basis of socio-cultural and religious teachings where male dominance is preponderant and women´s roles include domestic functions, child bearing and rearing as well as sexual obligations towards their partners.

The utilization of family planning commodities such as oral contraceptive pills, injectables and other methods independently reflects some measure of independence by the respondents and self-determination of fertility control especially considering that only 3.4% stated that spousal decision was the reason for contraceptive use [22]. According to data from the World Bank, contraceptive prevalence rate in Nigeria increased from 6% to 14% between 1990 and 2012 [23]. This rate has been hampered by factors ranging from unmet need for family planning to myths and erroneous perceptions in some parts of the country that contraceptives cause long term infertility [24]. Conversely, factors responsible for the increase include advocacy and political interventions to dispel these myths as well as health promotion activities by multiple stakeholders. Oral contraceptive pills were the most commonly used family planning method by the study participants with less than 5% utilizing contraception on account of their spouses. It has been reported that with increasing age, women are less likely to discuss contraceptive choices with their spouses and are probably able to achieve their fertility preferences [22].

Out-of-pocket payment constitutes a large chunk of health care financing in Nigeria which could have accounted for the respondents´ preference for public health institutions such as primary health care centers and secondary health care facilities (general hospitals). An assessment of the Nigerian healthcare system though inadequate in coverage has shown that public health care facilities are relatively affordable when compared with the private health sector on the basis that government is the major employer of skilled health manpower [25]. Respondents in this study also had a preference for physicians and nurses who are trained as skilled birth attendants despite a cultural belief in traditional birth attendants who also provide delivery services in most rural settings. A smaller age gap between spouses of less than 10 years was more favorable for respondents´ actions to maximize their own health as observed for autonomy indicators such as shared decision-making, choice of antenatal care provider and other reproductive decisions.

## Conclusion

Women´s reproductive independence has the potential of promoting utilization of reproductive health services thereby reducing maternal ill-health and mortality. Moreover, household joint decision-making and shared negotiation could result in higher male involvement and better maternal health.

### What is known about this topic

- Maternal and child mortality remains unacceptable in developing countries such as in sub-Saharan Africa;
- Health care seeking behavior of women is pertinent in the prevention and control of morbidity related to reproductive health;
- Utilization of reproductive health services can be influenced by women´s autonomy and decision-making capacity within the household.

### What this study adds

- A good number of women opined that their spouses should not be denied conjugal sex on any account;
- Spousal age difference less than 10 years was more favorable for autonomy indicators such as shared decision-making.
